# The corporate takeover of Alzheimer's treatment: a crisis in neurological autonomy?

**DOI:** 10.1055/s-0046-1817020

**Published:** 2026-03-11

**Authors:** Abelardo Q.C. Araujo

**Affiliations:** 1 Instituto de Neurologia Deolindo Couto, Rio de Janeiro RJ, Brazil.; 2 Universidade Federal do Rio de Janeiro, Faculdade de Medicina, Rio de Janeiro RJ, Brazil.

**Keywords:** Alzheimer Disease, Antibodies, Monoclonal, Health Care Sector, Professional Autonomy

## Abstract

The recent approval of monoclonal antibodies for the treatment of Alzheimer's disease represents a significant advancement in neurology. This accomplishment coincides with a worrisome trend: the increasing hegemony of large corporate healthcare organizations and commercial laboratory corporations in the supply of these new medications. This editorial examines how corporate influence undermines the traditional physician–patient relationship, diminishes neurologist autonomy, and signals a broader incursion into neurological practice by nonspecialists. The current situation in Brazil, compared with the United States and Europe, including the United Kingdom, is discussed, and strategies are proposed to maintain the integrity of neurological therapy amid growing corporate influence in the medical field.

## INTRODUCTION

The therapeutic landscape for Alzheimer's disease (AD) has undergone significant changes with the approval of anti-amyloid monoclonal antibodies (mAbs), such as lecanemab (Leqembi, Eisai Laboratórios Ltda.) and donanemab (Kisunla, Eli Lilly do Brasil Ltda.), which target amyloid beta (Aβ).

Lecanemab received conventional approval in the United States (US) in July 2023, following demonstration of clinical efficacy in the CLARITY AD trial, which showed a 27% reduction in deterioration on the Clinical Dementia Rating-Sum of Boxes (CDR-SB) scale after 18 months compared with placebo, 1 supporting the Aβ cascade hypothesis. Donanemab obtained the US Food and Drug Administration's (FDA) clearance in July 2024 for adults with mild cognitive impairment or mild dementia due to Alzheimer's disease. However, it is important to note that donanemab used a different primary outcome measure, the Integrated Alzheimer's Disease Rating Scale (iADRS) than lecanemab. When assessed by the CDR-SB scale for direct comparison, donanemab reduced cognitive decline by approximately 29% in the combined population. 2

These different outcome measures reflect evolving trial methodology but should not be interpreted as evidence of superior efficacy. The magnitude of clinical benefit appears comparable between the two agents when assessed by identical measures. These drugs represent the first disease-modifying therapeutics demonstrating statistically significant, though clinically modest, benefits in slowing cognitive deterioration. The absolute reduction averages 0.45 to 0.76 points on the CDR-SB scale over 18 months. These trials recruited carefully selected populations at early disease stages with documented amyloid and tau pathology, with limited representation of underrepresented ethnic and racial groups and patients with significant comorbidities, raising questions about the generalization of efficacy to diverse clinical populations.

This therapeutic revolution emerges with escalating corporate dominance in specialized medical care. Major healthcare companies and commercial laboratory chains are creating dedicated infusion facilities, directly targeting patients through popular media, and utilizing teams of neurologists as employed physicians. 3 This strategy drastically changes the traditional continuity of care, when patients sustained enduring connections with their neurologists, who navigated them through the intricate progression of neurodegenerative diseases.

## THE CORPORATE INFRASTRUCTURE CHALLENGE

The infusion of anti-amyloid monoclonal antibodies (mAbs) requires advanced infrastructure. Treatment consists of regular intravenous infusions (monthly for lecanemab, biweekly for donanemab) accompanied by mandatory magnetic resonance imaging (MRI) monitoring for amyloid-related imaging abnormalities (ARIA), which includes ARIA-E (vasogenic edema) observed in 12.6% of lecanemab patients and ARIA-H (microhemorrhages and hemosiderosis) in 17.3%. 1 Implementation involves substantial infrastructure investment, the designation of enterprise-wide project managers, interdepartmental cooperation among neurology, radiology, infusion services, and laboratory medicine, and the installation of comprehensive monitoring systems. 4 This complexity has created opportunities for corporate entities to position themselves as essential intermediaries.

Corporate-owned infusion centers offer comprehensive solutions, including fast access to therapy, integrated imaging services, and a team of employed physicians. Prominent commercial organizations are creating specialist centers, such as those described by Rosenbloom et al., 4 which require extensive infrastructure development. This paradigm may bridge infrastructure deficiencies, but it fundamentally transforms the physician–patient relationship. Patients, seduced by assertive direct-to-consumer marketing and media attention, are increasingly circumventing their established neurologists to pursue treatment at these corporate institutions. 5 The result is a disintegration of care, with pivotal treatment decisions made by clinicians who possess insufficient longitudinal awareness of the patient's clinical history, comorbidities, and psychosocial circumstances.

Furthermore, this corporate focus on pharmacological innovation risks marginalizing the holistic, multidisciplinary approach (speech therapy, physical rehabilitation, cognitive and social stimulation), which remains the only therapeutic option for most patients and forms the cornerstone of neurological practice across the entire disease spectrum. The concentration of capital and attention on anti-amyloid therapeutics risks diverting limited healthcare resources away from evidence-based, nondrug interventions—such as exercise programs, cognitive stimulation, and cardiovascular risk management—which remain fundamental to neurological practice and have demonstrated benefits across all stages of disease.

## LOSS OF NEUROLOGIST AUTONOMY

The corporate paradigm reduces neurologists from autonomous clinical decision-makers to employees within profit-oriented frameworks. This transition has a significant impact on professional autonomy and the quality of patient care. Corporate standards may emphasize efficiency and productivity over personalized evaluation.

The intricate decision-making involved in anti-amyloid medication necessitates a careful review of advantages versus the dangers of ARIA, especially in APOE ε4 carriers. This genotype substantially influences ARIA risk, with ARIA-E rates of 32.6% for lecanemab in APOE ε4 homozygotes compared to 5.4% in noncarriers. Notably, donanemab was not approved in Brazil for homozygous ε4 carriers, reflecting regulatory recognition of this risk stratification. Yet clinical judgment about individual risk–benefit ratios becomes subordinate to standardized corporate algorithms.

Furthermore, the economic incentives embedded in corporate healthcare may affect treatment recommendations. With Kisunla costing around US$ 32,000 for a 12 month supply, 6 and Leqembi at similar pricing levels, the financial stakes are substantial. Corporate companies may be motivated to optimize treatment initiation, thereby downplaying the associated hazards or alternative management approaches. The Coverage with Evidence Development requirement imposed by the Centers for Medicare & Medicaid Services (CMS) adds administrative complexities that favor large corporate organizations at the expense of independent practices. 7

## THE BRAZILIAN CONTEXT: UNIQUE CHALLENGES AND OPPORTUNITIES

Brazil faces unique challenges in implementing anti-amyloid therapy that differ substantially between the public and private healthcare sectors. Research carried out by Mattke et al. 8 indicates that Brazil lacks a national dementia strategy and a defined protocol for assessing cognitive issues, resulting in late, if any, diagnoses of dementia. The healthcare system's availability is significantly constrained, with anticipated wait times of almost 2 years for Alzheimer's evaluation and intervention, primarily due to a scarcity of dementia specialists (2.7 specialists per 100,000 population, compared to the G7 average of 11.27). 8 In the Brazilian Unified Health System (Sistema Único de Saúde, SUS, in Portuguese), significant regional disparities are evident. While older antidementia medications (donepezil, rivastigmine, galantamine, memantine) are available, more developed regions show higher utilization rates. 9

Thus, the SUS faces a dual challenge of limited access to specialists and insufficient financial capacity to support long-term management of high-cost therapies at scale, particularly given the modest clinical benefit, and significant infrastructure demands of anti-amyloid treatment. The concentration of specialist expertise and treatment capacity is geographically restricted to large urban centers.

The substantial infrastructure demands of anti-amyloid therapy—including requisite MRI facilities, genetic testing (APOE genotyping) capabilities, and specialized infusion infrastructure—are particularly challenging outside major urban centers, where these resources are dispersed or unavailable. This geographic concentration of treatment capacity paradoxically increases corporate opportunity while limiting equitable access. Recent epidemiological data indicate a significant disease burden, with substantial mortality and substantial per capita costs to healthcare systems. 10

The private sector, serving affluent and middle-class populations with greater access to infrastructure and specialists, will likely become the initial target for corporate infusion center development. At the same time, SUS faces mounting pressure to provide access without adequate resources, creating a two-tiered system that exacerbates existing inequalities. As international healthcare corporations enter underdeveloped economies, they introduce resources and infrastructure, but simultaneously risk instituting care paradigms that prioritize profit at the expense of professional autonomy and holistic patient care.

The critical locus of neurologist autonomy lies not primarily in administering infusions, but in the longitudinal clinical assessment, interpretation of advanced imaging studies, management of treatment-related adverse effects, genetic risk stratification, and modification of therapy based on individual patient response. When these functions are fragmented across corporate-employed physicians and centralized algorithms, the quality of individualized clinical care deteriorates.

## INTERNATIONAL COMPARISONS: LESSONS FROM DEVELOPED HEALTHCARE SYSTEMS

### United States

The US healthcare system has undergone a rapid corporatization of specialized medical treatment. The CMS, a federal agency within the US Department of Health and Human Services, issued a National Coverage Determination for FDA-approved monoclonal antibodies under Coverage with Evidence Development, requiring participation in registries, including the Alzheimer's National Registry for Treatment and Diagnostics, as well as various institutional registries. 7 This regulatory framework, whilst promoting evidence collection, has unintentionally enabled the proliferation of corporate infusion centers that can more readily navigate complex administrative demands than independent practitioners.

Prominent medical institutions have developed specialist programs, exemplified by the Dominantly Inherited Alzheimer's Disease (DiAD) clinic at Beth Israel Deaconess Medical Center. 11 Venture capital-backed enterprises are progressively establishing networks of infusion centers aimed exclusively at anti-amyloid therapy. These businesses employ assertive marketing tactics, eliminating the need for referrals from physicians and directly engaging patients through social media and targeted advertising. The American Academy of Neurology has created resources for its members; 12 nevertheless, their implementation is inconsistent across various care environments.

### Europe

European healthcare systems, which retain strong traditions of public provision, have demonstrated some resilience against corporate encroachment. However, pressures are increasing across both European Union member states and the United Kingdom. Austria's healthcare system, for example, shows that only 52.8 patients each year meet criteria for treatment at large memory clinics, 13 constituting roughly 10% of the patient population in these clinics, highlighting the selective nature of treatment eligibility and the infrastructure demands.

The United Kingdom's National Health Service (NHS) faces particular challenges in balancing access to innovation with cost management. Recent advances in digital prescriptions and healthcare delivery illustrate efforts to modernize while preserving public oversight. 14 The capacity of business entities to offer rapid access to breakthrough therapies creates a dual system that undermines the principle of equitable access to healthcare. 15

## THE BROADER CRISIS: SPECIALIST ENCROACHMENT

The corporate interference in anti-amyloid therapy illustrates a wider dilemma in neurology: the fragmentation of care when specialized therapies are administered outside longitudinal physician–patient relationships. While geriatricians and old age psychiatrists are recognized specialists in cognitive disorders—as explicitly affirmed by the Brazilian Academy of Neurology's position paper—the concerning trend is the episodic, corporate-mediated delivery of these complex immunotherapies. 17

The issue is not interdisciplinary collaboration per se, but rather the loss of continuity when specialized anti-amyloid therapy occurs outside the context of comprehensive neurological evaluation and long-term monitoring. When clinicians without specialized training in amyloid immunotherapy mechanisms, ARIA detection, and neuroimaging interpretation manage these therapies, patient safety is compromised.

The corporate model intensifies this fragmentation by creating episodic infusion-focused encounters separated from the comprehensive diagnostic and monitoring functions essential to safe therapy. Furthermore, practitioners trained primarily in neurosurgery but who function clinically as generalists should not be considered appropriate specialists for complex immunotherapeutic agents without additional training in neurodegeneration and in the management of amyloid-related imaging abnormalities. This fragmentation is particularly concerning in settings with weak regulatory oversight.

## PROPOSED SOLUTIONS AND RECOMMENDATIONS

### Strengthening professional governance

Neurological societies, including the Brazilian Academy of Neurology, must provide leadership in establishing guidelines for the administration of anti-amyloid medication. The position paper of the Brazilian Academy of Neurology about anti-amyloid treatments presents a framework for professional guidance. 16 This entails the creation of certification systems for treatment centers that emphasize continuity of care, mandate neurologist leadership, and ensure the implementation of suitable patient selection and monitoring methods. Implementation frameworks, such as those suggested by Rosenbloom et al., 4 should be modified to preserve physician autonomy.

### Regulatory reform

Governments must acknowledge the distinct issues presented by the proliferation of corporate healthcare. Regulations must require neurologist participation in the initiation and oversight of treatment, limit direct-to-consumer marketing of intricate neurological therapies, mandate transparency in corporate ownership and financial affiliations, and guarantee that employed physicians retain professional independence in clinical decision-making. The CMS Coverage with Evidence Development model 7 offers a framework, but requires modification to prevent corporate dominance.

The multiple sclerosis (MS) model offers an instructive precedent. While MS specialists similarly faced corporate infusion center development and complex treatment access algorithms, many MS neurology communities have successfully maintained high degrees of professional governance and clinical autonomy by establishing clear specialist guidelines, creating rigorous certification standards for treatment centers, and maintaining mandatory neurologist oversight of all treatment decisions. These MS-derived strategies offer a tested framework for maintaining neurological autonomy in Alzheimer's care.

Professional societies should establish rigorous certification standards (“quality seals”) for anti-amyloid treatment centers, with mandatory neurologist leadership, periodic external audits, transparent outcome reporting, and clear protocols for adverse event management. This would provide patients and insurance providers with evidence-based alternatives to corporate institutions while maintaining professional accountability.

### Strengthening public healthcare infrastructure

Brazil and other countries with limited access to specialists must allocate resources towards neurological training programs and enhance public healthcare infrastructure. Recent analysis reveals that investment in Alzheimer's disease treatment is alarmingly inadequate and insufficient to alleviate its impact. 10 Establishing centers of excellence inside public healthcare systems can offer alternatives to corporate care models while upholding professional standards. The discrepancies observed in Southern Brazil 9 underscore the urgent need for balanced infrastructure development.

### Collaborative care models

Instead of capitulating to corporate acquisition, neurologists should establish collaborative frameworks that maintain professional independence while fulfilling infrastructural requirements. Examples include neurologist-led consortia that distribute infrastructure expenses and collaborations with academic medical centers, such as those emerging in the US, 11 as well as integration with existing public healthcare systems, and telemedicine programs to extend specialist reach. Virtual care models 18 offer potential solutions for extending specialist expertise.

### Education and advocacy

Professional associations must inform both physicians and the public regarding the significance of continuity in neurological care. The educational programs of the American Academy of Neurology, 12 for example, offer a framework for professional development. Patients must acknowledge that while corporate centers provide convenience, the fragmentation of treatment may compromise long-term outcomes. Media campaigns should emphasize the importance of established physician–patient relationships in managing chronic neurodegenerative diseases.

### International collaboration

Due to the worldwide scope of pharmaceutical development and corporate healthcare growth, international cooperation is vital. The rapid development of antibodies for 2025 and subsequent years 19 will necessitate concerted efforts. Global neurological societies must collaborate on measures to preserve professional autonomy and ensure the quality of treatment. The establishment of worldwide standards for anti-amyloid treatment administration may help mitigate corporate pressure to prioritize efficiency over quality.

In conclusion, a victory for neurology should be on the horizon with the arrival of disease-modifying medicines for Alzheimer's disease. On the contrary, it risks becoming a cautionary tale about how corporate interests may hijack therapeutic innovation, undermining professional independence and causing healthcare to become disjointed. Particularly challenging are circumstances in nations like Brazil, where corporate exploitation is common due to a lack of infrastructure and specialists.

The next step is for healthcare systems, regulators, neurologists, and professional organizations to collaborate in addressing this problem. We must fight against the trend toward treating neurology like a commodity, with physicians coming and going in business settings. Because neurological diseases are so complex, patients need the care, continuity, and independent judgment of neurologists.

Neurology is at a crossroads; the choices made today will determine whether it remains distinct from other medical specialties and continues to focus on providing holistic, patient-centered care, or if it merges with corporate healthcare, prioritizing profit over professional quality. The integrity of our profession and the well-being of millions of patients who depend on our guidance as they confront neurodegenerative diseases are at stake. By learning from precedents in other specialties like MS neurology, and by implementing robust professional governance mechanisms, neurologists can ensure that this therapeutic advance benefits patients without compromising clinical autonomy, continuity of care, or the multidisciplinary approach that remains fundamental to neurological practice.
